# Redox, acid-base and clinical analysis of preterm and term neonatal lambs

**DOI:** 10.21451/1984-3143-2017-AR0054

**Published:** 2018-08-16

**Authors:** Liege Cristina Garcia Silva, Fernanda Machado Regazzi, Cristina Fátima Lúcio, Gisele Almeida Lima Veiga, Daniel Souza Ramos Angrimani, Claudia Barbosa Fernandes, Camila Infantosi Vannucchi

**Affiliations:** Department of Animal Reproduction, School of Veterinary Medicine and Animal Science.

**Keywords:** glutathione peroxidase, lung, neonate, superoxide dismutase, TBARS

## Abstract

During pregnancy, fetal lambs are exposed to low oxygen tension. Thus, an effective antioxidant mechanism is partially developed which sensitizes fetus to oxidative stress. Consequently, term and preterm neonates are susceptible to molecular and cellular injury caused by oxygen species (ROS). This study aimed to evaluate the development of antioxidant enzymes and oxidative profile of preterm (135 days of pregnancy) and term (145 days of pregnancy) neonatal lambs, correlating with clinical analysis. Preterm lambs had significantly (P ≤ 0.05) lower score of vitality (4.00 ± 1.10), bradycardia (99 ± 34 bpm) and bradypnea (13 ± 10 mpm). However, both groups were normothermic and euglycemic. Preterm group had low blood pH (7.07 ± 0.10) and both groups had hypercapnia, more severe in preterm group (85.52 ± 18.65 mmHg). In addition, premature newborns had lower pO^2^ (10.67 ± 5.65 mmHg) and SO^2^ (6.17 ± 5.85%) values. No significant difference (P ≥ 0.05) on antioxidant enzymes and oxidative stress were verified among experimental groups, although glutathione peroxidase negatively correlated with Apgar score, heart rate, SO^2^ and pO^2^. Our data show that preterm neonates are less adapted to the odds of labor and to overcome the immediate changes of extra-uterine life. Furthermore, we verified an influence of glutathione peroxidase in controlling oxidative stress, which highlights mature enzymatic mechanisms of cell redox, even in premature lambs.

## Introduction

The neonatal period represents an adaptive phase in which clinical outcome is inwardly related to physiological maturity. Neonatal mortality rate during the first weeks in lambs is estimated to be higher than to 15% ( Dwyer and Morgan, 2006 ). However, in premature lambs, such percentage can be much higher, resulting in significant economic losses to the sheep industry.

During pregnancy, fetal lambs are exposed to low oxygen tension (O^2^; Gitto *et al*., 2002 ). Consequently, the system of combating free radicals is partially developed and the imbalance between pro- and anti-oxidant systems sensitizes fetus to oxidative stress. With the onset of aerobic metabolism after birth, there is a significant increase in O^2^ consumption, which triggers the production of free radicals in the mitochondrial respiratory chain ( Vlessis and Mela-Riker, 1989 ). Of the total O^2^ consumed by cells, approximately 5% are metabolized into reactive oxygen species (ROS) such as superoxide anion (O^2-^), hydrogen peroxide (H^ 2^O^2^) and hydroxyl radical (OH^-^). Thus, the excessive production of ROS without an effective antioxidant mechanism is responsible for molecular and cellular injury to different body tissues, especially the brain and lung ( Gitto *et al*., 2002 ). The mechanism of oxygen toxicity is attributed to the direct or indirect action of free radicals in cellular components, leading to, for example, enzyme denaturation, disturbances in intracellular calcium homeostasis and cell death ( Freeman and Crapo, 1982 ; Rodrigues, 1998 ).

In premature human newborns, oxidative stress is even more severe than for term births ( Perrone *et al*., 2010 ). The premature infant is especially susceptible to ROS damage because adequate concentrations of antioxidants are absent at birth, due to low maternal-fetal placental transfer and endogenous production; and the ability to increase the antioxidant synthesis in response to hyperoxia is deficient ( Davis and Auten, 2010 ). Hypoxemic preterm neonates present high levels of plasma free radicals and protein oxidation metabolites at delivery and at seven days of life ( Buonocore *et al*., 2002 ).

The injuries caused by O^2^ free radicals are counteracted by enzymatic antioxidant defenses, such as superoxide dismutase (SOD), catalase and glutathione peroxidase (GPx) and non-enzymatic antioxidant defenses, for example, vitamins E, C, uric acid and bilirubin. Antioxidant profile of human newborns is marked by low levels of GPx, SOD, beta-carotene, riboflavin, riboproteinase, vitamin E, selenium, among other factors ( Gopinathan *et al*., 1994 ). In veterinary medicine, studies on antioxidant competence in the newborn sheep are yet to be performed.

In human fetus, there is a parallel of the antioxidant maturation pattern with the pulmonary surfactant system, with a 150% increase in SOD, GPx and catalase during the last 15% of gestation ( Davis and Auten, 2010 ). Therefore, we hypothesize that the redox system is related to acid-base balance after birth and premature lambs have both an antioxidant and clinical impairment compared to term neonates, which leads to the necessity of a specific reanimation protocol. Thus, the aims of this study were to evaluate the development of the antioxidant system at the end of gestation and the oxidative profile of preterm and term neonatal lambs, correlating with the clinical analysis immediately at birth.

## Materials and Methods

The present study complied with the ethical requirements for the use of animals in experiments, and was approved by the Bioethics Committee of the Faculty of Veterinary Medicine and Animal Science, University of São Paulo (protocol number 2039/2010).

### Animals and experimental design

A total of 12 newborn lambs of both genders were analyzed, divided into 2 groups according to gestational age at birth: preterm group (135 days of pregnancy; n = 6), term group (145 days of pregnancy; n = 6).

We used eight clinically healthy Santa Ines ewes, aged from 2 to 5 years, raised in an intensive system. Females were fed a commercial balanced ration, hay and mineral supplementation twice daily and water was provided *ad libitum*. Ewes were subjected to heat induction protocol with a single injection of 2.5 mg tromethamine dinoprost (Lutalyse^® ^, Pfizer) via IM. Natural matings with a single ram was conducted every 12 h during acceptance period. Therefore, the gestational period was considered 24 h after the first day of mating ( Jainudeen and Hafez, 2004 ). Ewes were clinically evaluated during pregnancy to verify fetal and maternal health status as well as the incidence of multiple pregnancies. In order to ensure labor induction exactly at 135 or 145 days of gestation, two sub-cutaneous applications of antiprogestogen aglepristone (Alizin^®^, Virbac) were made every 24 h at a dose of 10 mg/kg. The first application occurred 41 h prior to the estimated date of lambing. No drugs were used to artificially enhance fetal maturation.

At the beginning of the vaginal labor, females were continuously monitored and fetuses in fetal dystocia were excluded from this experiment. Immediately at birth, newborns were dried and vigorously rubbed at chest area. Then, oral and nasal cavities were gently aspirated with a urethral probe (Mark Med^®^) coupled to a secretion aspirator (Nevoni ^®^). Lambs were kept in an infant warmer (Fanem^®^, São Paulo, Brazil) with a radiant warmer (articulate reflector) and adjustable temperature at 25-30^o^C throughout the experiment.

### Clinical, acid-base and glucose neonatal analysis

Complete physical examination was performed immediately after birth through neonatal Apgar score of vitality adapted to lambs ( Vannucchi *et al*., 2012 ), with an additional separate analysis of cardiopulmonary auscultation, heart rate (HR) and respiratory rate (RR). Furthermore, lambs were evaluated for their rectal temperatures at the same time that the Apgar assessment was performed.

Immediately after clinical evaluation, arterial blood samples were collected by femoral artery puncture with heparinized sterile needles and syringes for hemogasometric analysis. We used i-STAT^®^ portable analyzer (Abbott) with the EG7+ cartridge, which provided the following data: pH, pO^2^ (oxygen pressure - mmHg), pCO^ 2^ (pressure of carbon dioxide - mmHg), TCO^2^ (total carbon dioxide - mmol/L), HCO^3-^ (bicarbonate - mmol/L), BE (base excess - mmol/L), SO^2^ (oxygen saturation - %), sodium (Na^+^ - mmol/L) and potassium (K^+^ - mmol/L).

Blood glucose concentration was determined in venous samples taken from jugular vein. A drop of blood was inserted in a glucometer (Advantage Monitor^®^, Roche Diagnostics) and the result was expressed as mmol/L.

### Antioxidant status analysis

The activity of the antioxidant enzymes superoxide dismutase (SOD) and glutathione peroxidase (GPx); and the marker of oxidative stress, thiobarbituric acid reactive substances (TBARS) were analyzed in serum samples. Immediately at birth, venous blood was collected from the jugular vein, centrifuged at 1500 xg during 10 min and the serum obtained was stored at -20^ o^C. In order to prevent enzyme degradation, the interval between sample collection and antioxidant analysis was not more than 30 days.

The measurement of thiobarbituric acid reactive substances (TBARS), aiming to access malondiadehyde levels, was made in accordance with a protocol first described by Ohkawa *et al*. (1979) . To precipitate proteins, 200 μl of serum and 400 μl of a 10% solution (v:v) of trichloroacetic acid (TCA 10%) were mixed and centrifuged (18,000 g for 15 min at 15°C). After centrifugation, 500 μl of the supernatant and 500 μl of 1% (v:v) thiobarbituric acid (TBA, 1%), in 0.05N sodium hydroxide in glass tubes were placed into a boiling water bath (100°C) for 10 min, and subsequently cooled in an ice bath (0°C) to stop the chemical reaction. TBARS were quantified by spectrophotometry, with a wavelength of 532 nm (Ultrospec 3300 Pro^®^, Amersham Biosciences). The results were compared to a standard curve previously made with a solution of malondiadehyde (MDA), using the value of 1.56 × 105 M−1 cm−1 as the MDA extinction coefficient ( Buege and Aust, 1978 ). The lipid-peroxidation index was described as nanograms of TBARS/ml of serum.

The GPx activity was measured by spectrophotometer (Ultrospec 3300 Pro^®^ , Amersham Biosciences) in a wavelength of 340 nm at 37°C for 100 min ( Nichi *et al*., 2007 ), followed by a reaction containing nicotinamide adenine dinucleotide phosphate (NADPH; 0.12 mM, 1 ml), oxidized glutathione (GSSGr; 0.25 U/ml, 20 ml) and GSH (1 mM, 100 ml). The results were expressed in U/ml.

The determination of serum SOD activity was performed according Flohe and Otting (1984) . The assay was performed in a spectrophotometer (Ultrospec 3300 Pro^®^ , Amersham Biosciences) at 550 nm and 25°C in a reaction medium containing cytochrome C (1 µm), xanthine (50 M), EDTA (100 µm) and sodium phosphate buffer (50 µm, pH 7.8). The results were expressed as U/ml.

### Statistical analysis

The results values were compared using SAS for Windows (SAS Institute Inc., Cary, NC, USA, 2000). The effect of gestational age groups (preterm *vs*. term) was determined using parametric (T test) and nonparametric (Wilcoxon) tests, according to the residue normality (Gaussian distribution) and variance homogeneity of each variable. Pearson and Spearman (parametric and no parametric variables, respectively) correlations were used to calculate the relationship between the variables studied in each variable group. Results are reported as untransformed means ± SEM. All values were considered significant at P < 0.05.

## Results

Clinical evaluation of lambs showed significant differences in Apgar scores, heart rate (HR) and respiratory rate (RR; Table 1 ). The preterm group had significantly lower vitality, HR (bradycardia) and RR (bradypnea). However, both groups were normothermic (preterm: 39.93 ± 0.42^o^C; term: 40.05 ± 0.36^o^C; P = 0.620) and euglycemic (preterm: 3.34 ± 1.88 mmol/L; term: 2.29 ± 0.82 mmol/L; P = 0.240).

**Table 1:** Mean ± SE of Apgar score, heart rate and respiratory rate in preterm and term groups.

Variable	Groups		P value
	Preterm	Term	
Apgar score (0-10)	4.00 ± 1.10	5.83 ± 0.98	0.012
Heart Rate (bpm)	99 ± 34	160 ± 50	0.034
Respiratory Rate (mpm)	13 ± 10	47 ± 18	0.002

At birth, preterm lambs had a low blood pH, indicating acidosis ( Table 2 ). Both groups had high pCO^2^ values, but there was a severe hypercapnia in the preterm group, indicating a respiratory imbalance. In addition, preterm newborns had hypoxemia, with significantly lower pO^2^ and SO^2^ values. There were no significant differences in TCO^2^ between groups ( Table 2 ). For both groups, the bicarbonate levels remained within normal ranges ( Vannucchi *et al*., 2012 ; Table 2 ). However, base excess was altered in both groups, indicating metabolic imbalance at birth ( Table 2 ).

**Table 2:** Mean ± SE of arterial blood gas values, sodium (Na^+^) and potassium (K ^+^) of preterm and term groups.

Variable	Groups		P value
	Preterm	Term	
pH	7.07 ± 0.10	7.27 ± 0.10	0.020
pCO^2^ (mmHg)	85.52 ± 18.65	45.3 ± 12.62	0.001
pO^2^ (mmHg)	10.67 ± 5.65	35.67 ± 16.18	0.011
SO^2^ (%)	6.17 ± 5.85	47.00 ± 28.48	0.016
TCO^2^ (mmol/L)	27.67 ± 1.97	22.00 ± 5.97	0.069
HCO^3-^ (mmol/L)	25.40 ± 1.98	20.73 ± 5.80	0.110
BE (mmol/L)	-3.50 ± 3.02	-5.00 ± 6.36	0.613
Na^+^ (mmol/L)	129.20 ± 40.26	143.20 ± 5.34	0.436
K^+^ (mmol/L)	4.72 ± 0.76	5.20 ± 2.08	0.611

pO^2^: oxygen pressure; pCO^2^: pressure of carbon dioxide; TCO ^2^: total carbon dioxide; HCO^3-^: bicarbonate; BE: base excess; SO^2^: oxygen saturation.

Although no difference was verified between groups, blood sodium concentration (Na^+ ^) was at low range (hyponatremia) only for preterm Group ( Table 2 ). On the other hand, blood potassium concentration (K^+^) remained at normal range for both groups ( Vannucchi *et al*., 2012 ; Table 2 ).

Regarding lambs oxidative profile, no difference (P > 0.05) on serum SOD, GPx and TBARS were verified among experimental groups ( Table 3 ).

**Table 3:** Mean ± SE of serum thiobarbituric acid reactive substances (TBARS), superoxide dismutase (SOD) and glutathione peroxidase (GPx) of preterm and term groups.

Variable	Groups		P value
	Preterm	Term	
TBARS (ng/ml)	113.75 ± 19.82	106.12 ± 15.68	0.464
SOD (U/ml)	0.41 ± 0.36	0.47 ± 0.14	0.703
GPx (U/ml)	14.19 ± 4.57	9.38 ± 3.04	0.058

There was a negative correlation (r = -0.914; P = 0.029) between glycemia and pH for the preterm group. Taking into account the oxidative profile of both groups, GPx results negatively correlated with Apgar score (r = -0.753; P = 0.004), HR (r = -0.576; P = 0.005), SO^2^ (r = -0.634, P = 0.026) and pO^2^ (r = -0.622, P = 0.030).

## Discussion

The Apgar score as an indicator of vitality in the immediate neonatal period may vary among domestic species ( Vannucchi *et al*., 2015b ). Lambs born at term in eutocia have Apgar score of approximately 6 immediately at birth ( Vannucchi *et al*., 2012 ). Thus, based on our results, it is clear that prematurity in sheeps entails the reduction of clinical vitality at birth, since all lambs had clinical depression. Additionally, the preterm group had significantly lower Apgar score, indicating an inadaptability condition of preterm lambs during transition to extra uterine life. As a compensatory response to moderate hypercapnia during normal labor, term neonates present increasing heart and respiratory rates ( Alonso-Spilsbury *et al*., 2005 ). In contrast, the preterm lambs in this study had low HR and RR at birth. Moreover, the premature group (135 days of pregnancy) had severe bradycardia and bradypnea. During neonatal period, the neurological control of cardiovascular system is incomplete due to partial sympathetic nervous activity of the myocardium. Thus, episodes of bradycardia are mostly derived from prolonged hypoxemia ( Grundy, 2006 ). Hence, bradycardia in neonates of the preterm group indicates increased hypoxic stress in comparison to the term group in which lamb respond to the hypoxic stress of labor by increasing their heart rate. These results highlight the low ability of premature lambs to overcome the challenge of lambing.

Premature lambs had blood acidosis, as well as severe hypercapnia. Similar results were obtained previously by Dani *et al*. (2009) , also for preterm lambs. The acid base imbalance that involves primarily changes of blood CO2 has a respiratory origin, causing respiratory acidosis or alkalosis ( Houpt, 1996 ). On the other hand, it is known that premature lambs are relatively tolerant to hypercapnia ( Strand *et al*., 2003 ). Thus, it is possible to infer that the severity of the acid-base imbalance is not primarily related to hypercapnia, but to the concurrent hypoxia. Therefore, we can assume that the main medical procedure to premature lamb’s resuscitation is to provide adequate pulmonary oxygenation.

In the present study, the preterm neonates had severe hypoxemia at birth, with low values of pO ^2^ and SO^2^ in comparison to full-term lambs ( Vannucchi *et al*., 2012 ). Neonatal blood pO^2^ is influenced by the concentration of inspired oxygen and respiratory rate ( Vaala *et al*., 2006 ). However, lambs became clinically depressed and bradypneic. Hence, low pO^2^ values indicated impairment of aerobic activity, which leaded to the onset of anaerobic metabolism. In addition, we observed a negative correlation between glycaemia and blood pH in the Premature Group, which means that consumption of glucose is necessary to achieve an blood acid-base balance, notably through the mobilization of energy reserves in order to increase respiratory and heart rate and compensate the initial hypoxia. Hence, it is extremely important to analyze the metabolic status of premature lambs and proceed to an adequate treatment of hypoglycemia whenever necessary.

The acid-base disturbance of non-respiratory origin should be evaluated by the deficit or base excess (BE) and the concentration of bicarbonate ions (HCO^3-^; Russell *et al*., 1996 ). Our results show that there is no depletion of serum bicarbonate and BE changes at birth, even in preterm lambs. Thus, blood gas alterations at birth reflected the hypoxia of lambing and not neonatal metabolic activity. Regardless of the fact that intrapartum hypoxia is considered physiologic, lambing assistance is of utmost importance, especially for premature lambs prone to more intense clinical depression and blood gas imbalance.

At late gestation, there is an increase of over 150% of the concentrations of SOD, GPx, catalase and glutathione reductase enzymes, parallel to functional maturation of human fetal lungs ( Davis and Auten, 2010 ). Consequently, there is a reduction of cellular lipid peroxidation ( Qanungo and Mukherjea, 2000 ). SOD catalyzes the reaction in which two O^2-^ molecules form H^2^O^ 2^ and O^2^. Subsequently, H^2^O^2^ is metabolized by reduced glutathione (GSH) in H^2^O and oxidized glutathione (GSSG), catalyzed by GPx ( Nichi *et al*., 2007 ). In human fetuses, during fetal-neonatal transition and in the immediate postpartum period, there is an increase in the activity of all enzymes that comprise the redox reaction of glutathione, especially GPx and glutathione transferase ( Vento *et al*., 2003 ). However, in our study, no significant difference in serum GPx and SOD was verified between premature and term lambs. Such results do not indicate improvement in the neonatal enzymatic antioxidant capacity during late gestational period in sheep. Nevertheless, additional experiments are needed using cytosol of erythrocytes to accurately detect neonatal antioxidant profile, because of the higher enzyme concentration compared to plasma ( Vento *et al*., 2003 ). On the other hand, Frosali *et al*. (2004) did not find significant differences in SOD and GPx between preterm and term infants, using the cytosol of erythrocytes as biological matrix.

In the present study, we demonstrated a negative correlation of GPx with the Apgar score, HR, pO^2^ and SO^2^. Based on such results, we can infer that neonates clinically depressed, in hypoxia and low O^2^ consumption produce high amounts of ROS that has to be counteracted by GPx. Hence, this enzyme is intensely consumed in such clinical situation. In fact, Vannucchi *et al*. (2015a) also attested the co-participation of GPx in the correction of neonatal acid-base imbalance in premature puppies. In addition, the action of GPx in reducing the rate of production of new free radicals also played a role in lamb’s vitality, as shown by Apgar score and HR.

Inder *et al*. (1994) documented increased lipid peroxidation and reduced GPx activity in preterm infants, as well as in low birth weight full-term babies with chronic lung disease. In our work, there was no difference in TBARS values between premature and term lambs. However, we cannot rule out local pulmonary increase in lipid peroxidation of premature sheep, as high production of free radicals is necessary to change serum concentration of TBARS ( Ballagipordany *et al*., 1991 ). Thus, it is possible that the local pulmonary measurement of TBARS, SOD and GPx over time in neonatal lambs can show differences in redox balance between preterm and term lambs.

To our knowledge, no previous reports have been performed to verify the effects of prematurity on lipid peroxidation and antioxidant enzymes, blood gases and clinical outcome of neonatal lambs. In summary, our data show that preterm neonates are less adapted to the odds of labor and to overcome the immediate changes of extra-uterine life. Furthermore, we verified an influence of glutathione peroxidase in controlling oxidative stress, which highlights mature enzymatic mechanisms of cell redox, even in premature lambs. This research offers understanding of the medical needs of prematurity, especially regarding acid-base adjustment and mechanical ventilation. However, further studies are necessary to better understand the local pulmonary antioxidant profile of ovine neonates and ultimately establish the mechanisms by which lambs neutralize the increase in reactive oxygen species.

Disclosure statement
